# Effect of magnesium sulfate on cerebral vasospasm in the treatment of aneurysmal subarachnoid hemorrhage: a systematic review and meta-analysis

**DOI:** 10.3389/fneur.2023.1249369

**Published:** 2023-11-10

**Authors:** Hanlin Zheng, Xiumei Guo, Xinyue Huang, Yu Xiong, Wen Gao, Chuhan Ke, Chunhui Chen, Zhigang Pan, Lichao Ye, Lingxing Wang, Weipeng Hu, Feng Zheng

**Affiliations:** ^1^Department of Neurosurgery, The Second Affiliated Hospital, Fujian Medical University, Quanzhou, China; ^2^Department of Neurology, The Second Affiliated Hospital, Fujian Medical University, Quanzhou, China

**Keywords:** magnesium sulfate, aneurysmal subarachnoid hemorrhage, cerebral vasospasm, delayed cerebral ischemia, secondary cerebral infarction, mortality, rebleeding, neurological dysfunction

## Abstract

**Introduction:**

The use of magnesium sulfate for treating aneurysmal subarachnoid hemorrhage (aSAH) has shown inconsistent results across studies. To assess the impact of magnesium sulfate on outcomes after aSAH, we conducted a systematic review and meta-analysis of relevant randomized controlled trials.

**Methods:**

PubMed, Embase, and the Cochrane Library were searched for relevant literature on magnesium sulfate for aSAH from database inception to March 20, 2023. The primary outcome was cerebral vasospasm (CV), and secondary outcomes included delayed cerebral ischemia (DCI), secondary cerebral infarction, rebleeding, neurological dysfunction, and mortality.

**Results:**

Of the 558 identified studies, 16 comprising 3,503 patients were eligible and included in the analysis. Compared with control groups (saline or standard treatment), significant differences were reported in outcomes of CV [odds ratio (OR) = 0.61, *p* = 0.04, 95% confidence interval (CI) (0.37–0.99)], DCI [OR = 0.57, *p* = 0.01, 95% CI (0.37–0.88)], secondary cerebral infarction [OR = 0.49, *p* = 0.01, 95% CI (0.27–0.87)] and neurological dysfunction [OR = 0.55, *p* = 0.04, 95% CI (0.32–0.96)] after magnesium sulfate administration, with no significant differences detected in mortality [OR = 0.92, *p* = 0.47, 95% CI (0.73–1.15)] and rebleeding [OR = 0.68, *p* = 0.55, 95% CI (0.19–2.40)] between the two groups.

**Conclusion:**

The superiority of magnesium sulfate over standard treatments for CV, DCI, secondary cerebral infarction, and neurological dysfunction in patients with aSAH was demonstrated. Further randomized trials are warranted to validate these findings with increased sample sizes.

## 1. Introduction

Aneurysmal subarachnoid hemorrhage (aSAH) is caused by the rupture of intracranial aneurysms and has a high morbidity rate of 6–9 per 100,000 persons and a mortality rate of approximately 35% (1). Previous data has revealed that more than 30% of patients with aSAH die within the first few days to weeks after initial bleeding, and most survivors experience long-term neurological dysfunction (2). The leading causes of disability and death are post-aSAH complications, including cerebral vasospasm (CV), delayed cerebral ischemia (DCI), secondary cerebral infarction, and rebleeding (3).

Although calcium antagonists such as nimodipine are commonly used to prevent CV, DCI, secondary cerebral infarction, and rebleeding in patients with aSAH (1), magnesium sulfate may provide an alternative for preventing such complications. A few studies have shown that magnesium sulfate is considered safe in treating aSAH (4); the higher the serum magnesium concentration, the lower the probability of CV (5). Hypomagnesemia can be observed in approximately 38% of patients upon admission (6), suggesting that serum magnesium concentrations may be closely related to CV after aSAH. Although several randomized controlled trials (RCTs) have recently evaluated the prophylactic use of magnesium sulfate in patients with aSAH, whether magnesium sulfate can improve the prognosis of aSAH remain controversial (6–13). Therefore, we performed a systematic review and meta-analysis to determine whether magnesium sulfate treatment could reduce the risk of CV and other poor outcomes in patients with aSAH.

## 2. Methods

### 2.1. Search criteria

We conducted this systematic review and meta-analysis according to the PRISMA guidelines (14). PubMed, Embase, and the Cochrane Library were searched for relevant articles written in English that were published from database inception to March 20, 2023. Full-text RCTs that compared the incidence of CV, DCI, secondary cerebral infarction, rebleeding, neurological dysfunction, and mortality between magnesium sulfate and control groups (saline or standard treatment) after aSAH in published studies were included. Case reports, comments, non-randomized studies, editorials, protocols, letters, guidelines, and animal studies were excluded. Unpublished studies were excluded from the analysis. The databases were searched using the appropriate MeSH terms and thw keywords “magnesium sulfate” and “subarachnoid hemorrhage.” We also searched the titles, abstracts, and subject headings of all potentially relevant articles. The reference lists of all included articles and review papers were carefully reviewed to obtain additional publications.

### 2.2. Outcome measures

We assessed the effects of magnesium sulfate and saline/standard treatment on poor patients outcomes, including CV, DCI, secondary cerebral infarction, rebleeding, neurological dysfunction, and mortality. The primary outcome of this meta-analysis was CV, which was defined as recurrence of aSAH symptoms after improvement of aSAH symptoms or increased velocity of cerebral blood flow, as detected by angiography and/or transcranial Doppler (3). The secondary outcomes included DCI (defined as symptoms or signs of neurological deficits relieved within 24 h), secondary cerebral infarction (defined as symptoms or signs of neurological deficits that persisted for more than 24 h or low-density lesions confirmed on head computed tomography (CT) with corresponding new symptoms or signs) (3), rebleeding, neurological dysfunction and mortality.

### 2.3. Statistical analysis

Review Manager software version 5.4 (The Cochrane Collaboration,2020; Nordic Cochrane Centre, Copenhagen, Denmark) for statistical analysis. Dichotomous data were summarized as odds ratios (ORs) and 95% confidence intervals (CIs). Continuous data were displayed as mean differences (MDs) and standard deviations (SDs). Where appropriate, SDs were calculated based on the reported standard errors. Cochran’s *Q* test was used to assess study heterogeneity. An *I*^2^ > 50% or *p* < 0.1 was said to represent significant heterogeneity. Due to the heterogeneity in treatment duration and definitions of neurological dysfunction between the included studies, a meta-analysis of random effects was utilized to pool the data for more robust results (15). Categorical variables were analyzed using the dominant ORs, and continuous variables using MDs. Additionally, a meta-analysis of random effects was used to combine the results of the original studies. If there was significant heterogeneity, a sensitivity analysis using the leave-one-out method was carried out to evaluate the robustness of the results and check if any single study included in the meta-analysis might have a significant effect on the final results. To achieve this test, data for each study was removed and meta-analyses were then recalculated for the remaining studies, so that the impact of the removed data on the overall study can be ascertained. The Cochrane Collaboration Tool was used to assess bias. *p*-values <0.05 were considered statistically significant.

## 3. Results

### 3.1. Literature search results

**Figure 1:**
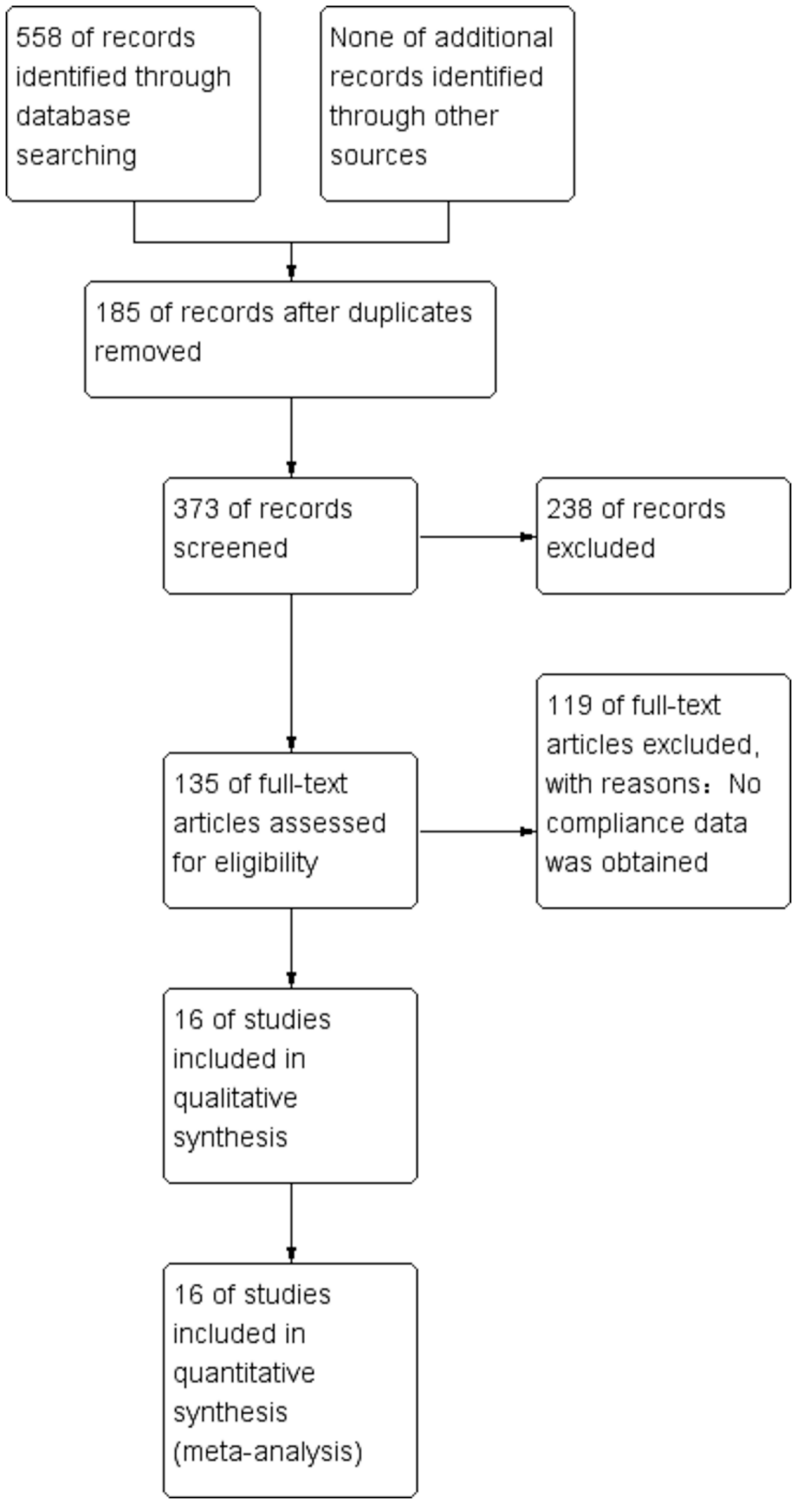
Literature search scheme.

We identified 558 studies across the databases reporting magnesium sulfate in aSAH, of which 373 published studies were obtained after removing duplicate records. After screening the titles and abstracts, 238 unrelated studies were excluded. The full texts of the remaining 135 studies were reviewed; eventually, 16 studies (3, 5–13, 16–21) with a total of 3,503 participants (1,764 and 1,739 in the magnesium sulfate and control groups, respectively) comparing the incidence of related complications in patients with aSAH treated with magnesium sulfate and control groups were included in the analysis (Figure 1). Eight studies (7, 8, 11, 12, 17–20) were conducted in Europe, six (3, 6, 9, 10, 16, 21) in Asia, one (13) in the Americas, and one (5) in Australia. The treatment duration in the 16 studies (3, 5–13, 16–21) was 10–21 days, and the follow-up time of most studies (5–7, 9–13, 16, 17, 19, 21) was more than 3 months. The baseline characteristics and the primary and secondary outcome indicators of each study are shown in Table 1.

**Table 1:** Characteristics of the included studies.

Studies	Patients (*n*)		Gender		Average age, y		CV		DCI		SCI		Mortality		Rebleeding		Neurological dysfunction	
	MG	CG	Male	Female	MG	CG	MG	CG	MG	CG	MG	CG	MG	CG	MG	CG	MG	CG
Akdemir et al. (16)	40	43	32	51	53.4	53.9	10	14	10	14	NA	NA	8	6	NA	NA	NA	NA
Boet et al. (10)	23	22	8	37	NA	NA	NA	NA	6	12	NA	NA	3	3	NA	NA	NA	NA
Bradford et al. (5)	79	78	NA	NA	55.8	56.6	40	50	NA	NA	NA	NA	NA	NA	NA	NA	NA	NA
Dorhout et al. (7)	604	596	362	838	57.0	57.0	NA	NA	NA	NA	NA	NA	91	85	NA	NA	NA	NA
Huenges et al. (17)	107	102	44	165	53.7	55.7	NA	NA	NA	NA	NA	NA	NA	NA	0	1	53	51
Kunze et al. (18)	54	53	41	66	50.0	52.0	NA	NA	NA	NA	0	9	NA	NA	NA	NA	9	23
Leijenaar et al. (8)	307	309	175	441	56.2	57.9	NA	NA	81	75	NA	NA	NA	NA	NA	NA	NA	NA
Muroi et al. (11)	31	27	43	15	52.1	53.6	12	10	3	6	3	6	4	6	NA	NA	4	4
Takeuchi et al. (12)	12	13	11	14	60.8	57.1	1	8	1	7	NA	NA	NA	NA	NA	NA	NA	NA
van den Bergh et al. (19)	139	144	99	184	54.8	54.4	NA	NA	22	36	63	67	27	31	NA	NA	NA	NA
Veyna et al. (13)	20	16	10	26	46.0	51.0	6	5	6	5	NA	NA	4	2	NA	NA	6	5
Westermaier et al. (20)	54	53	66	41	50.0	52.0	36	45	12	27	12	27	6	10	NA	NA	9	15
Wong et al. (21)	30	30	18	42	58.0	62.0	11	17	7	13	NA	NA	4	5	NA	NA	NA	NA
Wong et al. (6)	169	158	119	208	57.0	57.0	42	29	42	29	17	18	22	28	NA	NA	42	29
Yamamoto et al. (9)	35	35	23	47	59.1	59.5	NA	NA	NA	NA	5	9	1	1	NA	NA	NA	NA
Zhang et al. (3)	60	60	62	58	43.5	42.9	4	12	3	10	2	8	5	8	4	5	3	11
Total	1,764	1,739	1,113	2,233	NA	NA	162	190	193	234	102	144	175	185	4	6	126	138

y, years; CV, cerebral vasospasm; DCI, delayed cerebral ischemia; SCI, secondary cerebral infarction; MG, magnesium sulfate group; CG, control group (saline/standard treatment); NA, not available.

### 3.2. Analysis of the quality of eligible studies

**Figure 2:**
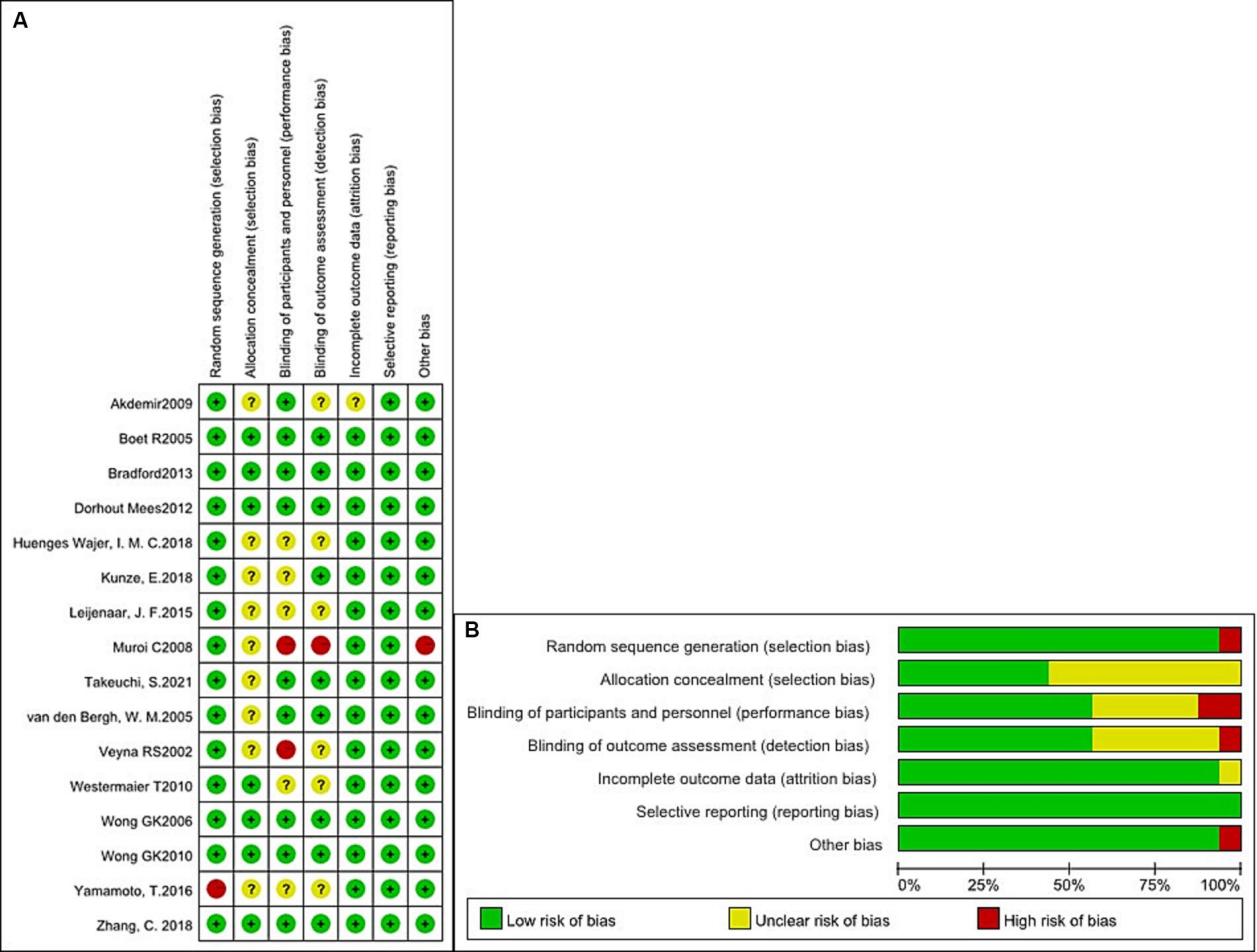
Quality analysis of the studies. **(A)** Summary of the risk of bias in all included studies. **(B)** Risk of bias graph: judgments about each risk of bias item presented as percentages across all included studies.

We evaluated the qualification standard for the 16 included studies using the Cochrane Collaboration tool. Quality analysis of the studies is shown in Figures 2A,B. All studies (3, 5–13, 16–21) described randomization methods, and seven studies (3, 5–7, 10, 13, 20) had sufficient allocation concealment. Regarding the blinding method, except for one study (11) with a single-blinded design, the remaining studies were double-anonymized (3, 5–10, 12, 13, 16–21). Finally, no study has selectively reported these results.

### 3.3. Outcomes

#### 3.3.1. CV

**Figure 3:**
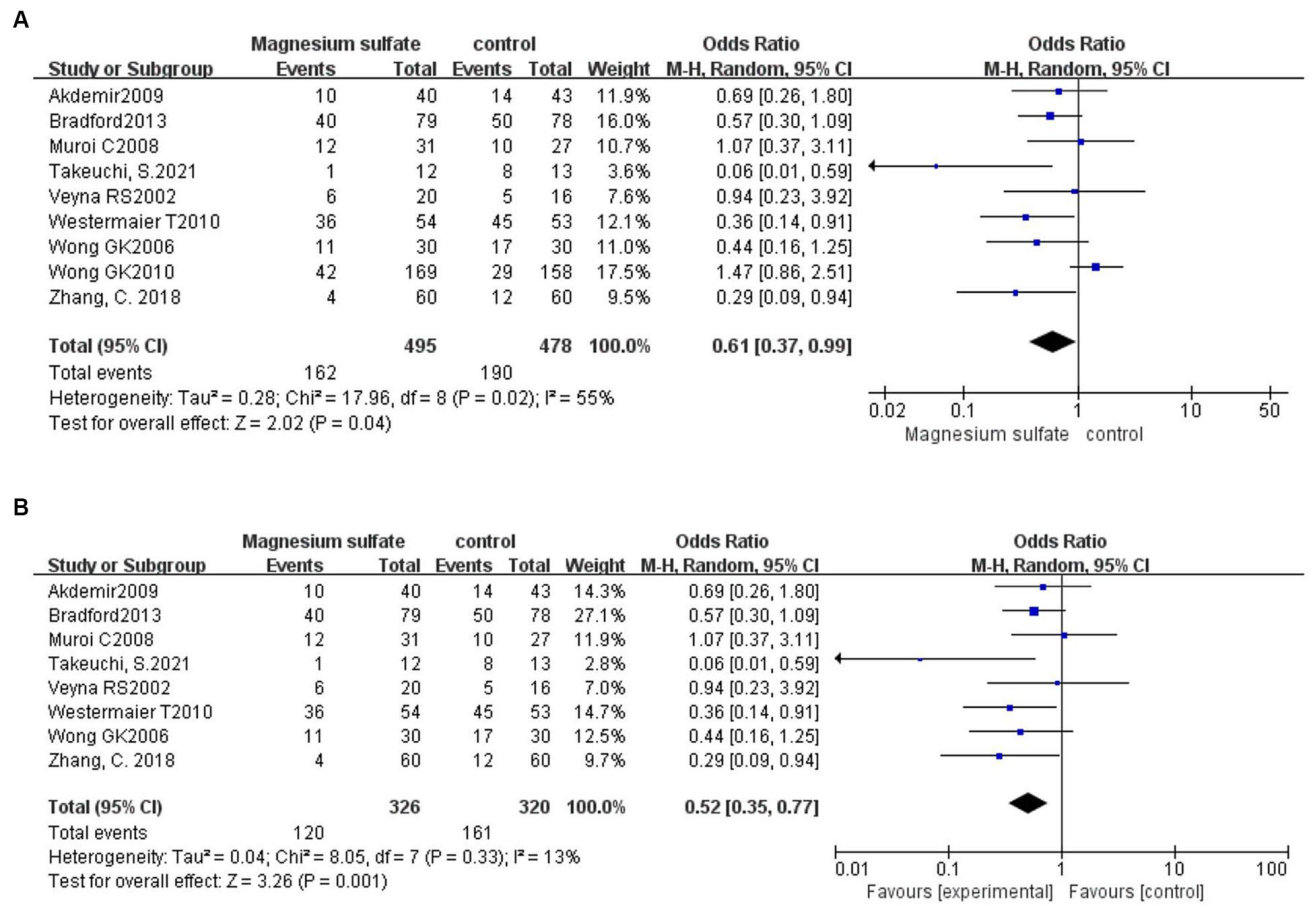
Forest plots showing the occurrence of cerebral vasospasm between magnesium sulfate and the control group before **(A)** and after **(B)** sensitivity analysis.

A total of 973 patients in nine studies (3, 5, 6, 11–13, 16, 20, 21) were evaluated for CV after aSAH. The results showed that compared with patients in control groups, the incidence of CV in aSAH patients treated with magnesium sulfate was significantly lower [OR = 0.61, *p* = 0.04, 95% CI (0.37–0.99)] (Figure 3A). Owing to significant heterogeneity (*p* = 0.02, *I*^2^ = 55%), we used sensitivity analysis to evaluate the robustness of the results. After excluding a trial by Wong et al. (6), heterogeneity was significantly decreased (*p* = 0.33, *I*^2^ = 13%), with no changes to the significant difference in favor of magnesium sulfate [OR = 0.52, *p* = 0.001, 95% CI (0.35–0.77)] (Figure 3B).

#### 3.3.2. DCI

**Figure 4:**
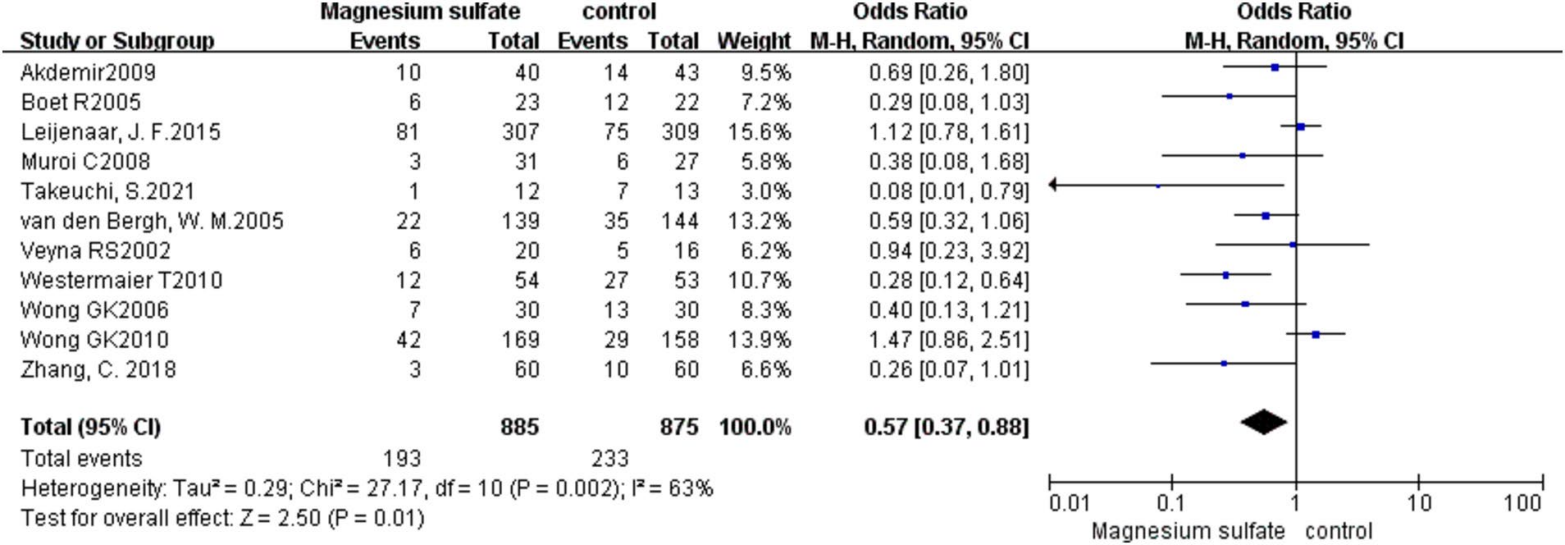
Forest plot showing the occurrence of delayed cerebral ischemia between magnesium sulfate and the control group.

DCI was recorded in 11 studies (3, 6, 8, 10–13, 16, 19–21) with 1,760 patients. Compared with the control group, the incidence of DCI in the magnesium sulfate group was significantly lower [OR = 0.57, *p* = 0.01, 95% CI (0.37–0.88)] (Figure 4). After sensitivity analysis, no exact source of heterogeneity was detected.

#### 3.3.3. Secondary cerebral infarction

**Figure 5:**
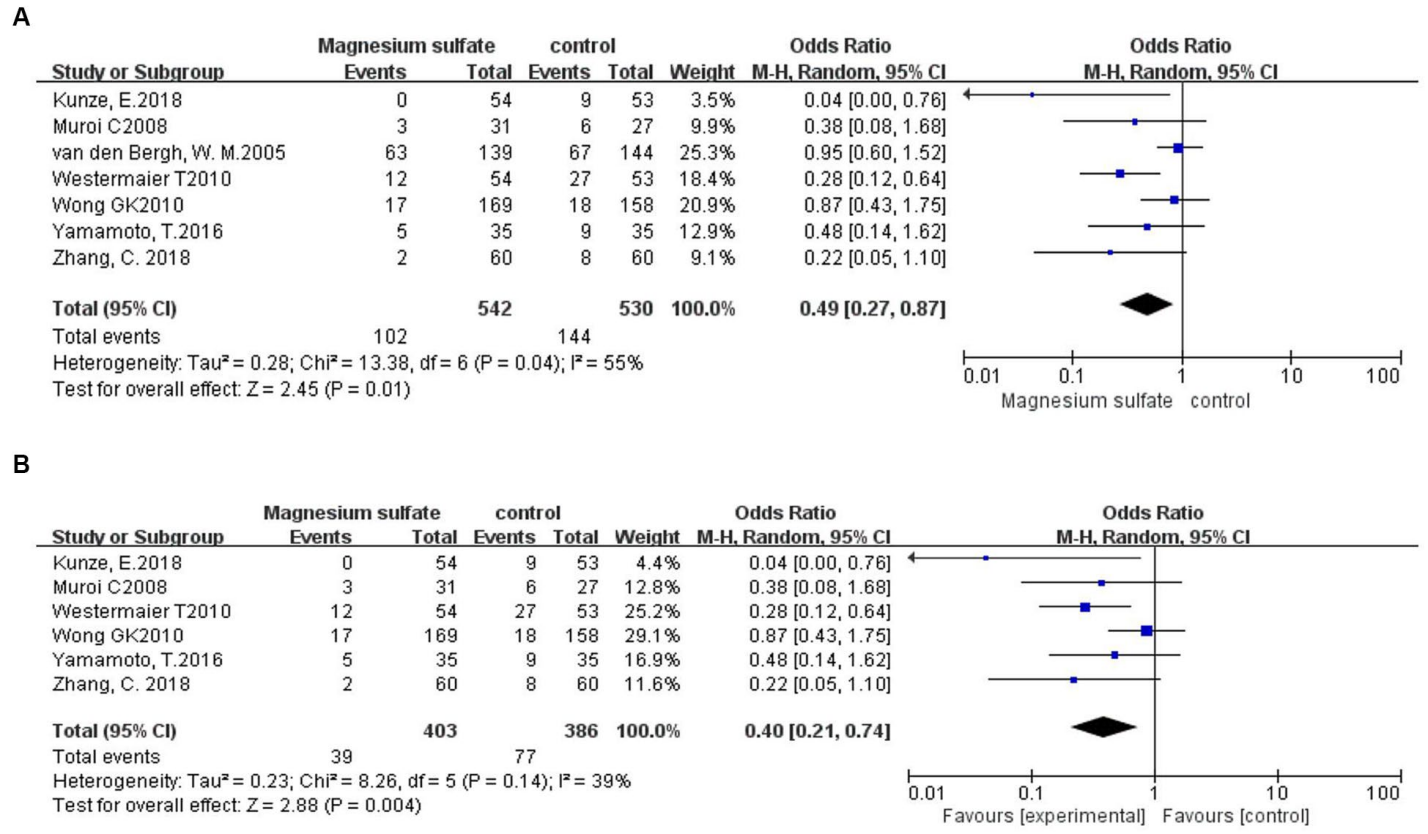
Forest plots showing the occurrence of secondary cerebral infarction between magnesium sulfate and the control group before **(A)** and after **(B)** sensitivity analysis.

Seven studies (3, 6, 9, 11, 18–20) assessed 1,072 patients with secondary cerebral infarctions. Compared with the control group, the incidence of secondary cerebral infarction in the magnesium sulfate group was reduced [OR = 0.49, *p* = 0.01, 95% CI (0.27–0.87)] (Figure 5A). Owing to significant heterogeneity (*p* = 0.04, *I*^2^ = 55%) across the studies, we used sensitivity analysis to evaluate the robustness of the results. After excluding a trial by van den Bergh et al. (19), heterogeneity was significantly decreased (*p* = 0.14, *I*^2^ = 39%), with no changes to the significant difference in favor of magnesium sulfate [OR = 0.40, *p* = 0.004, 95% CI (0.21–0.74)] (Figure 5B).

#### 3.3.4. Mortality

**Figure 6:**
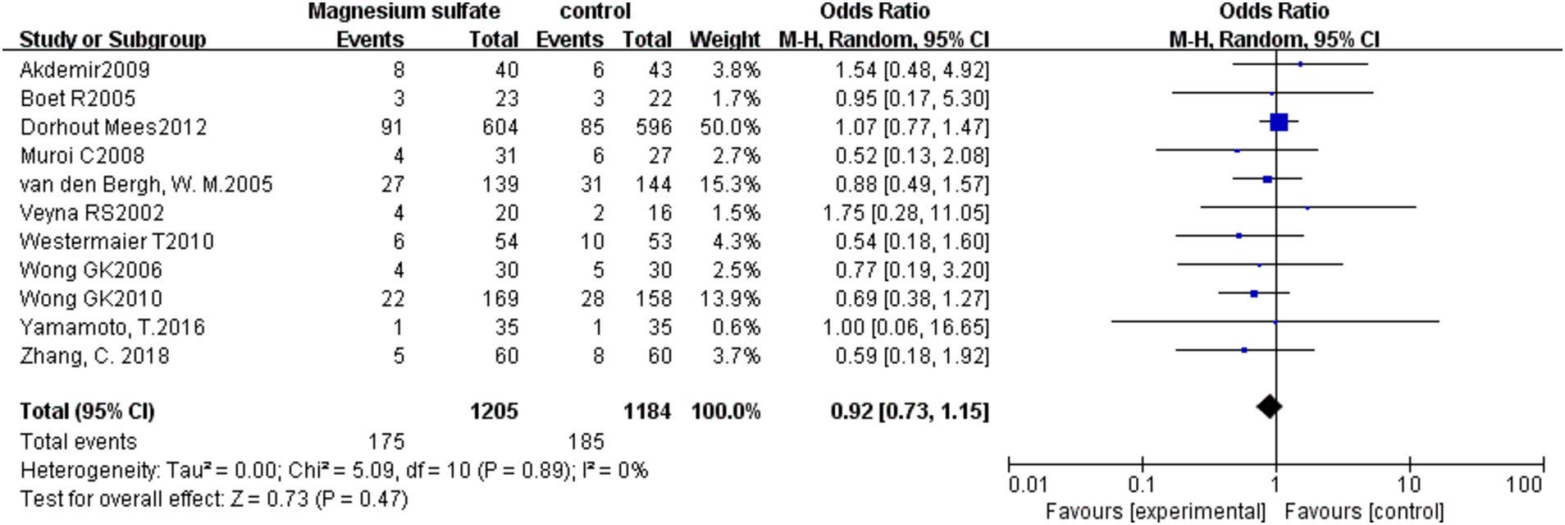
Forest plot showing the mortality between magnesium sulfate and the control group.

The pooled results from 11 studies (3, 6, 7, 9–11, 13, 16, 19–21) with 2,389 patients showed that there was no significant difference in mortality between the magnesium sulfate group and the control group in the treatment of aSAH [OR = 0.92, *p* = 0.47, 95% CI (0.73–1.15)] (Figure 6).

#### 3.3.5. Rebleeding

**Figure 7:**
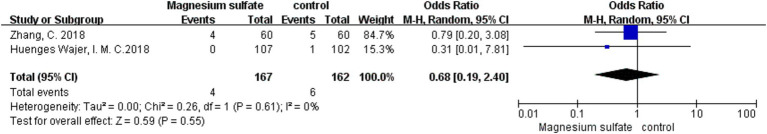
Forest plot showing the occurrence of rebleeding between magnesium sulfate and the control group.

Among the 16 studies, only two (3, 17) documented data on rebleeding in 329 patients. The pooled data showed no significant difference in the occurrence of rebleeding between the magnesium sulfate and control groups [OR = 0.68, *p* = 0.55, 95% CI (0.19–2.40)] (Figure 7).

#### 3.3.6. Neurological dysfunction

**Figure 8:**
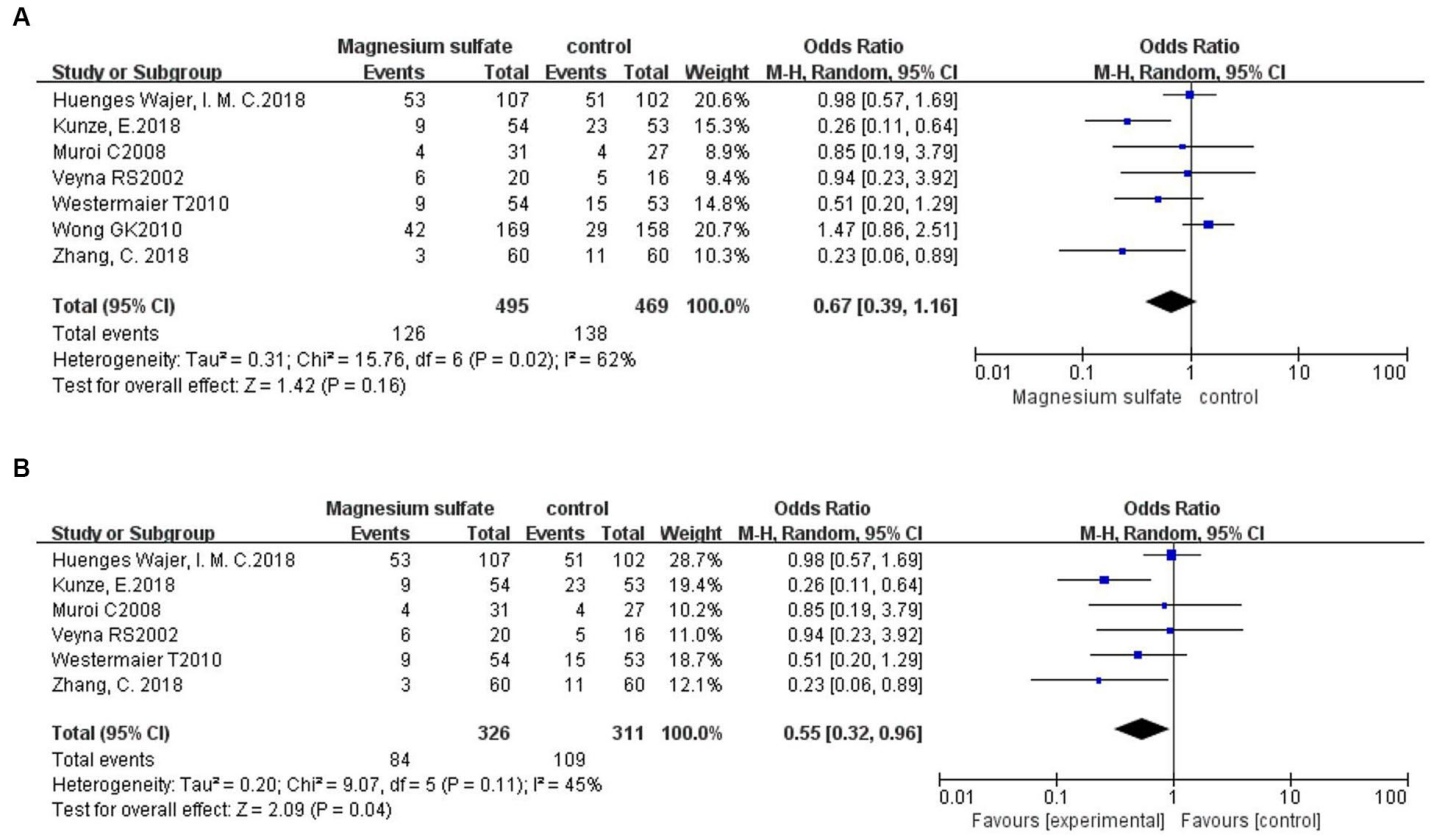
Forest plots showing the occurrence of neurological dysfunction between magnesium sulfate and the control group before **(A)** and after **(B)** sensitivity analysis.

Seven studies (3, 6, 11, 13, 17, 18, 20) evaluated neurological dysfunction in 964 patients. There was no significant difference in neurological dysfunction between the magnesium sulfate and control groups [OR = 0.67, *p* = 0.16, 95% CI (0.39–1.16)] (Figure 8A). Further sensitivity analysis was performed due to the significant heterogeneity (*p* = 0.02, *I*^2^ = 60%). After excluding a study by Wong et al. (6), the heterogeneity was significantly decreased (*p* = 0.11, *I*^2^ = 45%) with a significant difference detected in favor of magnesium sulfate [OR = 0.55, *p* = 0.04, 95% CI (0.32–0.96)] (Figure 8B).

## 4. Discussion

CV, DCI, secondary cerebral infarction, and rebleeding mainly occurs within 2 weeks after aSAH onset, in which the incidence of CV and DCI are 30%–90% and 17%–40%, respectively; the high incidence of complications suggests the need for prevention (2, 3, 7). Although the calcium channel blocker nimodipine is currently the first-line treatment for aSAH (1), some studies have suggested that magnesium sulfate, which is inexpensive, has similar or even better efficacy in the treatment of aSAH; its safety and tolerability have been confirmed in the treatment of eclampsia (4, 11, 16).

**Figure 9:**
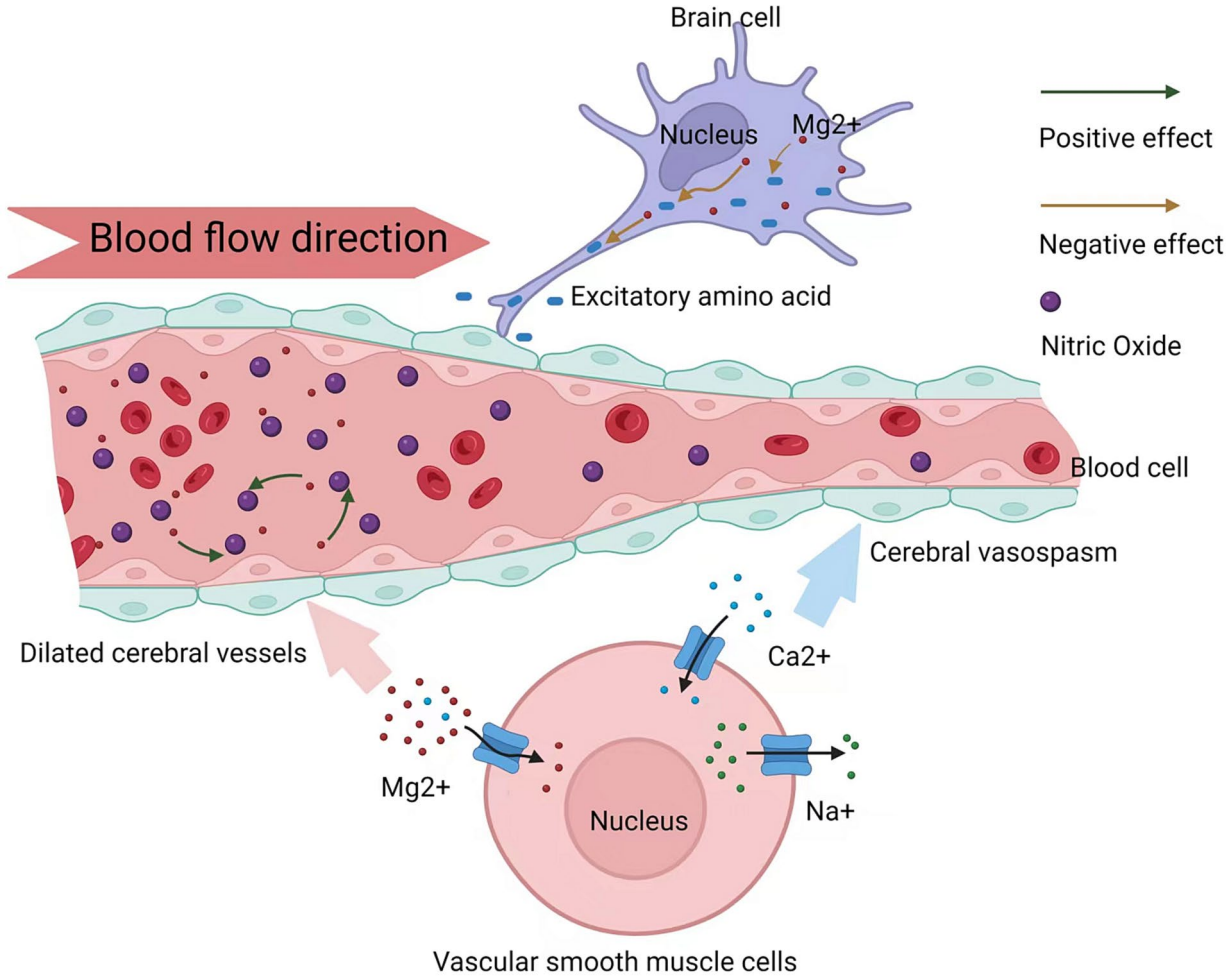
Mechanism plot of magnesium sulfate in preventing poor outcomes of aneurysmal subarachnoid hemorrhage.

Theoretically, magnesium ions may exert neuroprotective effects in preventing and improving CV and other complications in patients with aSAH via three mechanisms. First, as natural calcium channel antagonists, magnesium ions block the influx of calcium ions, inhibiting blood vessel contraction and preventing CV. Second, after the onset of aSAH, the role of the vasoconstrictor nitric oxide is weakened; however, magnesium ions can enhance the role of nitric oxide, thereby preventing CV. Third, magnesium sulfate prevents brain cells from releasing excitatory amino acids and acts as a vasodilator in cerebral arteries (3, 21, 22) (Figure 9).

Nine studies (3, 5, 6, 11–13, 16, 20, 21) recorded the influence of magnesium sulfate on CV. The incidence rates of CV in the magnesium sulfate group and in the control group were 32.7% and 39.7%, respectively, demonstrating that magnesium sulfate significantly reduced the incidence of CV. However, due to the presence of substantial heterogeneity among the included studies, a sensitivity analysis was performed, showing that the significant difference in magnesium sulfate treatment remained unchanged. This was inconsistent with previous studies (6, 13, 16, 21), which may have been due to the different infusion days and maintenance doses of soluble magnesium sulfate in the included studies.

Regarding DCI, 11 studies (3, 6, 8, 10–13, 16, 19–21) recorded relevant outcomes. The incidence rates of DCI in the magnesium sulfate and control groups were 21.8% and 26.6%, respectively, showing a significant difference in favor of magnesium sulfate, consistent with most previous studies (3, 19, 20, 23). This may be because magnesium ions can act as vasodilators to reduce the occurrence of DCI by improving vasospasms and ischemia tolerance (20). Due to significant heterogeneity, the results need to be interpreted with caution.

Seven studies (3, 6, 9, 11, 18–20) reported secondary cerebral infarction outcomes. The incidence rates of secondary cerebral infarction in the magnesium sulfate group and in the control group were 18.8% and 27.2%, respectively, indicating a considerable difference between the two groups. Due to the presence of significant heterogeneity, another sensitivity analysis was performed. After excluding the study by van den Bergh et al. (19), the significant difference observed with magnesium sulfate treatment did not change. This may be because magnesium sulfate can reduce the area of cerebral infarction by improving the CV and shortening the total ischemic time, thereby preventing secondary cerebral infarction (13).

Eleven (3, 6, 7, 9–11, 13, 16, 19–21) of the 16 studies analyzed patient mortality. The mortality rates in the magnesium sulfate and control groups were 14.5% and 15.6%, respectively, with no significant differences detected between the two groups. Limited by the relatively small sample sizes, further studies are warranted to determine whether magnesium sulfate reduces mortality in patients with aSAH.

Two studies (3, 17) reported the effect of magnesium sulfate on rebleeding. The incidence of rebleeding in the magnesium sulfate and control groups was 2.4% and 3.7%, respectively, with no significant difference between the two groups, indicating that magnesium sulfate may not reduce rebleeding in patients with aSAH. Due to the scarcity of available data, future studies with larger sample sizes are needed to verify this finding.

Neurological dysfunction was recorded in seven (3, 6, 11, 13, 17, 18, 20) studies. There was no significant difference in the incidence of neurological dysfunction between the two groups initially; however, there was substantial heterogeneity. After conducting the sensitivity analysis, there was a significant difference in favor of magnesium sulfate, indicating that patients in the magnesium sulfate group had better neurologic and functional outcomes. However, in the included studies, the definitions of neurological dysfunction differed and multiple assessment scales were used, including the National Institutes of Health Stroke Scale (3), the modified Rankin scale (6, 17), and the Glasgow Outcome Scale (6, 11, 13, 18, 20), which may have affected the validity of our results.

Despite recent publications regarding magnesium sulfate in the treatment of aSAH, whether magnesium sulfate can improve CV and other poor outcomes remains controversial (3, 5–7, 12, 18–20). Some studies (3, 12, 18–20) have reported that magnesium sulfate treatment for aSAH can reduce the incidence of CV, DCI, secondary cerebral infarction, and neurological dysfunction, while other studies (5–7) have concluded that magnesium sulfate treatment for aSAH is not beneficial. In the present study, compared to standard treatments, magnesium sulfate was shown to reduce the incidence of these post-aSAH outcomes. Further studies are warranted to verify these findings.

Our study had some limitations. First, in the included studies, the infusion days and maintenance doses of magnesium sulfate were different, which may have affected the results of our analysis. Second, although the number of patients exceeded 3,000, the number of patients with some outcomes was relatively small, especially for the rebleeding outcome, making it difficult to obtain statistical differences. Third, the length of follow-up varied between the included studies, subacute and late complications were more likely to be reported in studies with longer follow-up periods.

In conclusion, the present meta-analysis provides evidence supporting the superiority of magnesium sulfate over standard treatments for reducing the occurrence of CV, DCI, secondary cerebral infarction, and neurological dysfunction in patients with aSAH. Limited by the differences between the studies and the relatively small sample sizes included, no significant differences were observed in the outcomes of rebleeding and mortality between magnesium sulfate and standard treatment groups. Further randomized trials with larger sample sizes are required to confirm these findings.

## Data availability statement

The original contributions presented in the study are included in the article/supplementary material, further inquiries can be directed to the corresponding authors.

## Author contributions

HZ, XG, XH, and YX: drafting the original manuscript and interpretation of the data. ZP and FZ: critical revision of the manuscript. LW and LY: conception and design of the study. WG and CK: literature search. CC: data extraction. WH: drafting the figures and tables. All authors contributed to the article and approved the submitted version.
